# Paget’s disease of the breast: Our 20 years’ experience

**DOI:** 10.3389/fonc.2022.995442

**Published:** 2022-11-22

**Authors:** Lorenzo Scardina, Alba Di Leone, Stefano Magno, Antonio Franco, Ersilia Biondi, Alejandro Martin Sanchez, Sabatino D’Archi, Damiano Gentile, Alessandra Fabi, Riccardo Masetti, Gianluca Franceschini

**Affiliations:** ^1^ Breast Unit, Department of Women, Children and Public Health Sciences, Fondazione Policlinico Universitario Agostino Gemelli IRCCS, Rome, Italy; ^2^ Breast Unit, IRCCS Humanitas Research Hospital, Milan, Italy; ^3^ Precision Medicine Breast Unit, Scientific Directorate, Department of Women, Children and Public Health Sciences, Fondazione Policlinico Universitario Agostino Gemelli IRCCS, Rome, Italy

**Keywords:** Paget’s disease, breast cancer, breast-conserving surgery, mastectomy, invasive cancer

## Abstract

**Introduction:**

Paget’s disease (PD) represents 1%–3% of all breast cancers and mostly occurs in postmenopausal women. Multiple studies have confirmed that breast-conserving surgery (BCS) followed by radiotherapy is a safe option for patients with *in situ* or invasive PD, ensuring local control and survival rates similar to those achieved with mastectomy.

**Materials and methods:**

We retrospectively analyzed 115 patients affected by PD treated in our institution between January 2000 and May 2021. Median age at diagnosis was 60 years and median follow-up was 82 months; 69 patients were treated with BCS and 46 were treated with modified radical mastectomy or skin-sparing mastectomy.

**Results:**

At histological examination, 59 patients (59/115, 51.0%) had an underlying invasive carcinoma; in 11 patients (11/115, 9.0%), only PD was found. In 45 patients (45/115, 40.0%), only noninvasive cancer was found. Nine patients (9/115, 7.8%) developed a local recurrence, 7 patients (7/115, 6.0%) are alive with distant metastasis, and 10 patients (10/115, 8.6%) died.

**Discussion:**

In our series, no statistically significant differences were shown between PD alone, PD associated with *in situ* cancer, and PD with invasive cancer, regardless of the surgical procedure. BCS followed by radiotherapy appears to be an effective and safe option for patients with PD.

**Conclusion:**

PD is a rare form of breast cancer and, in half of the cases, is associated with an invasive carcinoma. Separating our sample into three subgroups based on tumor histology, there were no significant differences in terms of LC, DFS, and OS rate in patients treated with different types of surgery. This study presents some limitations due to its retrospective nature and being confined to a single institution.

## Introduction

Paget’s disease (PD) is an uncommon breast lesion associated with cancer characterized by heterogeneous clinical features, such as erythema, nipple scaling, eczematous rash, and skin ulceration. The symptoms more often reported are bleeding, pain, and itching. Described by Sir James Paget in 1874 (1), it accounts for only 1%–3% of all breast cancers (2–4) mostly in postmenopausal women.

Prognosis is determined by the presence or absence of underlying invasive cancer.

It is widely accepted that the intradermal Paget cells originate from an underlying ductal cancer, but the histogenesis and pathogenesis of Paget cells remains controversial. The current theory argues that luminal lactiferous ductal epithelial cells give rise to Paget cells, which migrate into the epidermis (5–7). Microscopically, typical Paget cells with pale, abundant cytoplasm and hyperchromatic nuclei are found in the epidermal layer.

Due to the rarity of this breast pathology, there is still an ongoing debate on the most appropriate treatment. Over the past decades, various randomized trials have found breast-conserving surgery (BCS) followed by radiotherapy to be an acceptable or even preferable alternative to mastectomy for patients with early-stage *in situ* or invasive PD (8), with local control and survival rates similar to those achieved with mastectomy (9–12).

Based on current standards, in all patients affected by PD undergoing mastectomy or conservative surgery for an invasive carcinoma, sentinel lymph node biopsy (SLNB) should be performed (13).

The prognosis of these patients depends primarily on the presence of an underlying invasive component (4). The presence of an underlying cancer, usually an invasive carcinoma, results in a worse prognosis related to the stage of malignancy (14).

The largest series of PD in literature is the one of Yufeng Yao and colleagues (15) made up of 5,398 patients with a 10-year follow-up period. All the other series in the last decade consist of less than 100 cases of PD.

In this paper, we retrospectively analyzed 115 patients affected by PD and treated at our institution between January 2000 and May 2021. The aim of the present study is to describe our experience and to outline the best plan of treatment.

## Materials and methods

All data were collected from our database at the Fondazione Policlinico Universitario Agostino Gemelli IRCCS of Rome, Italy. We performed a retrospective review of 115 consecutive cases of PD of the breast treated during the period 2000–2021 and presenting with or without a palpable mass. The patients consisted of 111 women and 4 men. The median age at diagnosis was 60 years (range, 32–88 years). The median follow-up time was 82 months (range, 2–230 months).

Each patient was discussed in a multidisciplinary meeting with pathologists, surgeons, and radiologists from our Breast Unit, in order to select the most appropriate surgical treatment, on the basis of international breast cancer guidelines and careful clinical and appropriate imaging assessment of each patient. A clinical examination and radiological imaging study with ultrasound, digital mammography, and magnetic resonance imaging were performed preoperatively. After primary surgery, all cases were evaluated at a multidisciplinary meeting to select systemic therapy. Patients’ characteristics and pathologic features are detailed in **Table 1**.

**Table 1:** Characteristics and clinicopathological features of 115 patients with confirmed Paget’s disease.

	Number (%)/Median (range)
**Sex**	
Female	111 (96.5%)
Male	4 (3.5%)
Age (years)	60 (32–88)
**Side**	
Right	49 (42.6%)
Left	66 (57.4%)
Underlying palpable tumor	35 (30.5%)
**Pathologic**	
PD alone	11 (9.0%)
PD + invasive carcinoma	59 (51.0%)
PD + noninvasive carcinoma	45 (40.0%)
**Histotype**	
- Invasive ductal	52 (45.2%)
- Invasive lobular	7 (6.0%)
- *In situ* ductal	45 (39.0%)
**Grading**	
-1	12 (10.5%)
-2	32 (27.8%)
-3	71 (61.7%)
Ki67 (%)	26 (0–90%)
**Stage**	
- pTis	56 (48.7%)
- pT1	25 (21.7%)
- pT2	21 (18.3%)
- pT3	3 (2.6%)
- pT4	10 (8.7%)
- N0	57 (49.5%)
- N1	25 (21.7%)
- N2	6 (5.2%)
- N3	3 (2.6%)
- Multifocal	33 (28.7%)
**Biological subtypes**	
- Luminal-like	17 (28.8%)
- HER2-enriched	34 (57.7%)
- Triple negative	8 (13.5%)
**Post-operative treatment**	
- Radiotherapy	71 (61.7%)
- Hormone therapy	33 (28.7%)
- Chemotherapy	35 (30.4%)

Patients were deemed candidates for nipple-areola excision alone only when a complete removal of the disease was feasible, while a BCS and SLNB were chosen for small and unifocal *in situ* or invasive carcinomas and no clinically positive lymph nodes.

Among the patients not suitable for BCS, 29 were treated with radical mastectomy and 17 patients were treated with skin-sparing/skin-reducing mastectomy.

Complete axillary dissection was performed in 32 patients with positive sentinel node (**Table 2**).

**Table 2:** Detailed surgical treatment of 115 patients with confirmed Paget’s disease.

Type of surgical treatment	Number (%)
**Breast surgery**	
- BCS	69 (60.0%)
- Mastectomy	46 (40.0%)
**Axillary staging**	
- No axillary staging	25 (21.7%)
- SLNB	58 (50.4%)
- ALND	32 (27.8%)

BCS, breast-conserving surgery; SLNB, sentinel lymph node biopsy; ALND, axillary lymph node dissection.

Radiation therapy was performed in all patients treated with BCS (69/115, 60.0% of the sample), while 35 patients (35/115, 30.4%) were treated with adjuvant chemotherapy.

Data were collected in order to evaluate oncological outcomes: local recurrence (LR), disease-free survival (DFS), and overall survival (OS).

### Statistical analysis

Results are expressed as means with standard deviations and median with ranges. Statistical analysis was performed using the SPSS (version 24.0 for Windows). Fisher exact test was used for comparison of categorical variables. A *p*‐value equal to or less than 0.05 was considered statistically significant. Estimates of OS, DFS, and LR were produced by cumulative incidence, using the Kaplan–Meier method. Multivariate analysis was not performed due to the limited number of events.

## Results

At histologic examination, the majority of patients (59/115, 51.0%) had an underlying invasive carcinoma; in 11 patients (11/115, 9.0%), only PD was found. In 45 patients (45/115, 40.0%) only noninvasive cancer was found. Thirty-five patients (35/115, 28.0%) presented with a palpable breast mass, and all patients with a palpable mass were found to have an invasive cancer. Forty-nine (49/115, 42.6%) cases of PD have been identified in the right breast and 66 (66/115, 57.4%) have been identified in the left breast, and the diagnosis of breast carcinoma with or without PD was confirmed histologically in all cases with preoperative biopsy or cytology.

Sixty-nine patients (69/115, 60.0%) were treated with BCS, while 46 patients (46/115, 40.0%) underwent conservative (nipple/skin sparing) or radical mastectomy.

### Oncological outcomes

With a median follow-up of 83 months (range, 2–230 months), nine patients (9/115, 7.8%) developed a first recurrence of disease in the treated breast. All recurrences in the breast had an invasive component. Seven patients (7/115, 6.0%) presented with distant metastasis. Overall, 10 patients (10/115, 8.6%) died, all presented with recurrences (**Table 3**).

**Table 3:** Oncological outcomes of 115 patients.

Total events	Breast-conserving surgery		Mastectomy	
	Invasive disease	Noninvasive disease	Invasive disease	Noninvasive disease
**Local Recurrence** 9 (/115, 7.8%)	4/29	2/40	2/30	1/16
**Metastasis** 7 (/115, 6.0%)	1/29	1/40	5/30	0/16
**Exitus** 10 (/115, 8.7%)	3/29	3/40	3/30	1/16

**Figure 1:**
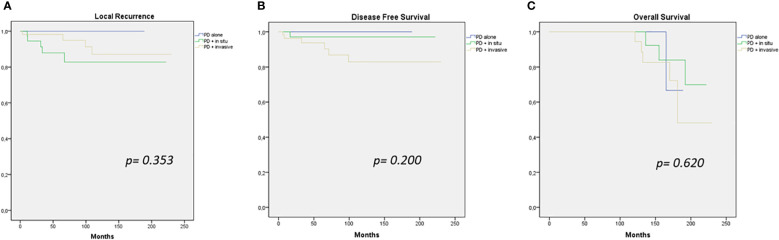
Local recurrence **(A)**, disease-free survival **(B)**, and overall survival **(C)** curves (PD alone – PD + *in situ* – PD + invasive cancer).

There was no significant difference in terms of LC, DFS, and OS between patients affected by PD alone, PD with *in situ*, and PD associated with invasive cancer (*p* = 0.353, *p* = 0.200, and *p* = 0.620, respectively) (**Figure 1**).

**Figure 2:**
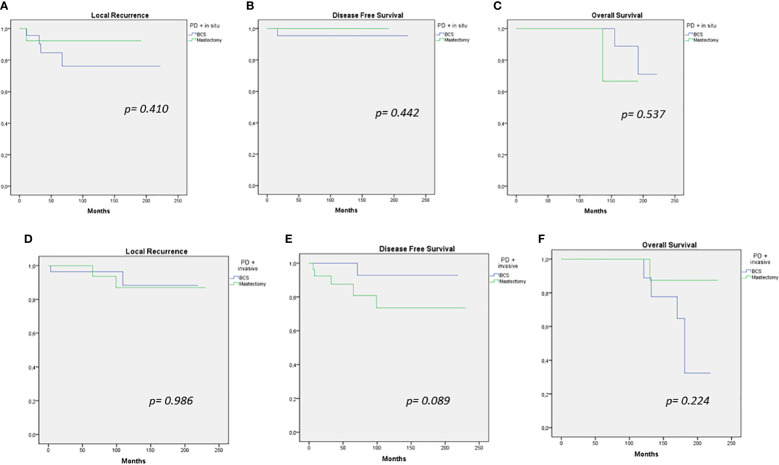
Local recurrence, disease-free survival, and overall survival curves for breast-conserving therapy versus mastectomy in PD + *in situ* **(A–C)** and PD + invasive cancer **(D–F)**.

Comparison of oncological outcomes (LC, DFS, and OS) of patients treated with different types of surgery (BCS or mastectomy) was performed, separating our sample into two subgroups based on tumor histology (PD + *in situ*, PD + IC). There were no significant differences in the LC, DFS, and OS rate between the subgroups (**Figure 2**).

## Discussion

This study presents a retrospective analysis of 115 patients affected by PD, presenting to a single institution over a 20-year period. PD is a very rare form of breast cancer. Previous studies on patient’s survival with PD have reported controversial results. According to Wu and colleagues (16), the prognosis was better for patients affected by PD in association with invasive breast cancer compared with those affected by breast cancer alone, while Ortiz-Pagan and colleagues (17) found that PD might have a negative effect on survival. Moreover, the OS rates between patients with PD associated with invasive breast cancer and patients with invasive breast cancer alone were similar.

In our retrospective analysis, we found no significant difference in oncological outcomes between patients affected by PD alone, PD with *in situ*, or PD associated with invasive cancer. This may be in part due to the fact that most patients in this series (95/115) have received adjuvant treatments (82.6%). In addition, half of the patients in our study (61/115) were operated on between 2015 and 2021 and therefore had a short follow-up period. Magnetic resonance imaging (MRI) seems more sensitive that mammography or breast ultrasound alone in terms of its ability to identify small lesions especially in young women or to detect retroareolar tumor tissue (18, 19). In patients with multifocal or multicentric lesions in association with PD, mastectomy was the standard of care. Although mastectomy has long been regarded as standard therapy for PD with or without associated breast cancer, the use of BCS is oncologically safe (20). Contraindications for BCS are extended microcalcification or multicentric cancer and poor cosmetic results. According to several studies, BCS is an effective treatment for selective patients affected by PD (21–23). Polgar and colleagues and Dixon and colleagues reported high LR rate after BCS (4, 24), but it was seen after surgery only, with no radiotherapy. Recent studies suggest that in selected patients with PD, BCS followed by radiotherapy was associated with significant survival benefits (8, 10). By now, it has been established that BCS and adjuvant radiotherapy for patients with PD are an effective local treatment strategy compared with mastectomy (15). In relation to survival analysis, no statistically significant difference was observed in our study in patients treated with different types of surgery (BCS or mastectomy). BCS in association with radiotherapy can be considered the therapeutic gold standard, except for extensive microcalcification or multicentric cancer and poor cosmetic results. We can confirm that surgical treatment of PD is based on parenchymal disease like typical breast cancer (15). Nowadays, the role of SLNB in the management of PD remains controversial (25). National Comprehensive Cancer Network (NCCN) guidelines encourage axillary staging for PD with an underlying invasive breast cancer, although axillary evaluation is not considered necessary for PD in association with DCIS treated with BCS (26). However, almost all patients with PD will have an underlying malignancy. In the study of Sukumvanich and colleagues (27), 90.0% of patients with PD had an associated invasive carcinoma and 26.0% of patients affected with PD only presented on the final histologic examination an invasive carcinoma, despite the appropriate presurgical workup and imaging. According to our experience, SLNB should be considered at the time of surgical treatment also in patients with PD in the event when MRI findings, in association with core biopsy, are suspicious for underlying cancer or positive lymph nodes. To date, this is one of the largest single-center series of PD with a long period of follow-up; nevertheless, it presents some limitations due to its retrospective nature and being confined to a single institution.

## Conclusion

PD is a rare form of breast cancer. In 30.0% of cases, it may present with a concomitant mass within the breast, and in at least half of the cases, it is associated with an invasive carcinoma; therefore, the correct treatment choices depend on the underlying staging and pathological features of the disease. The present study should be interpreted in the context of its limitations. As regards local treatment, for selected patients, BCS in association with adjuvant radiotherapy appears to be an effective and safe option; in our experience, SLNB should be considered in all PD patients with an underlying invasive carcinoma and/or suspicious axillary findings. At follow-up, the recurrences and survival rates of PD seem to be comparable to those of breast carcinoma overall. These conclusions cannot be definitive and clearly more prospective randomized trials are needed in order to establish a standard of care for these patients.

## Data availability statement

The raw data supporting the conclusions of this article will be made available by the authors, without undue reservation.

## Ethics statement

Ethical review and approval was not required for the study on human participants in accordance with the local legislation and institutional requirements. Written informed consent for participation was not required for this study in accordance with the national legislation and the institutional requirements.

## Author contributions

Conceptualization: LS and EB. Methodology: LS and MS. Validation: LS and SD’A. Formal analysis: AnF and EB. Investigation: AnF, SD’A, and MS. Data curation: AnF and EB. Writing—original draft: LS, EB, and DG. Writing—review and editing: LS and SM. Visualization: GF, SM, and AL. Supervision: RM, GF, and AlF. Project administration: RM and SM. All authors contributed to the article and approved the submitted version.

## Conflict of interest

The authors declare that the research was conducted in the absence of any commercial or financial relationships that could be construed as a potential conflict of interest.

## Publisher’s note

All claims expressed in this article are solely those of the authors and do not necessarily represent those of their affiliated organizations, or those of the publisher, the editors and the reviewers. Any product that may be evaluated in this article, or claim that may be made by its manufacturer, is not guaranteed or endorsed by the publisher.
